# A neuromuscular exercise programme versus standard care for patients with traumatic anterior shoulder instability: study protocol for a randomised controlled trial (the SINEX study)

**DOI:** 10.1186/s13063-017-1830-x

**Published:** 2017-02-28

**Authors:** Henrik Eshoj, Sten Rasmussen, Lars Henrik Frich, Inge Hvass, Robin Christensen, Steen Lund Jensen, Jens Søndergaard, Karen Søgaard, Birgit Juul-Kristensen

**Affiliations:** 1 Department of Sports Science and Clinical Biomechanics, University of Southern Denmark, Odense, Denmark; 2 Department of Clinical Medicine, Aalborg University, Aalborg, Denmark; 3 Orthopaedic Surgery Research Unit, Aalborg University Hospital, Aalborg, Denmark; 4 Department of Orthopaedics and Traumatology, Odense University Hospital, Odense, Denmark; 5 Shoulder Sector, Orthopedic Department, South-West Jutland Hospital, Esbjerg, Denmark; 6 Musculoskeletal Statistics Unit, The Parker Institute, Bispebjerg and Frederiksberg Hospital, Copenhagen, Denmark; 7 Shoulder Sector, Orthopaedic Department, Aalborg University Hospital, Farsoe, Denmark; 8 Research Unit of General Practice, Institute of Public Health, University of Southern Denmark, Odense, Denmark; 9 Institute of Occupational Therapy, Physiotherapy and Radiography, Department of Health Sciences, Bergen University College, Bergen, Norway; 10 Department of Sports Science and Clinical Biomechanics, University of Southern Denmark, Campusvej 55, 5230 Odense M, Denmark; 11 Odense Quality of Life Research Center, Department of Haematology, Odense University Hospital, Odense, Denmark

**Keywords:** Shoulder, Dislocation, Instability, Neuromuscular exercise, Physiotherapy, Nonoperative treatment

## Abstract

**Background:**

Anterior shoulder dislocation is a common injury and may have considerable impact on shoulder-related quality of life (QoL). If not warranted for initial stabilising surgery, patients are mostly left with little to no post-traumatic rehabilitation. This may be due to lack of evidence-based exercise programmes. In similar, high-impact injuries (e.g. anterior cruciate ligament tears in the knee) neuromuscular exercise has shown large success in improving physical function and QoL. Thus, the objective of this trial is to compare a nonoperative neuromuscular exercise shoulder programme with standard care in patients with traumatic anterior shoulder dislocations (TASD).

**Methods/design:**

Randomised, assessor-blinded, controlled, multicentre trial. Eighty patients with a TASD will be recruited from three orthopaedic departments in Denmark. Patients with primary or recurrent anterior shoulder dislocations due to at least one traumatic event will be randomised to 12 weeks of either a standardised, individualised or physiotherapist-supervised neuromuscular shoulder exercise programme or standard care (self-managed shoulder exercise programme). Patients will be stratified according to injury status (primary or recurrent). Primary outcome will be change from baseline to 12 weeks in the patient-reported QoL outcome questionnaire, the Western Ontario Shoulder Instability Index (WOSI).

**Discussion:**

This trial will be the first study to compare the efficacy and safety of two different nonoperative exercise treatment strategies for patients with TASD. Moreover, this is also the first study to investigate nonoperative treatment effects in patients with recurrent shoulder dislocations. Lastly, this study will add knowledge to the shared decision-making process of treatment strategies for clinical practice.

**Trial registration:**

ClinicalTrials.gov, identifier: NCT02371928. Registered on 9 February 2015 at the National Institutes of Health Clinical Trials Protocol Registration System.

**Electronic supplementary material:**

The online version of this article (doi:10.1186/s13063-017-1830-x) contains supplementary material, which is available to authorized users.

## Background

Traumatic anterior shoulder dislocation (TASD) is a common injury [1], especially within the second and third decades of life in young, active individuals [2], with a yearly incidence rate in the general population reported to be from 11.2 to 26.2 per 100,000 persons [3–7]. Following a first-time TASD, the risk for recurrent dislocations is high due to pathophysiological changes in the shoulder joint [8] with mean risk rates estimated to vary between 39% and 67% [4, 9, 10]. Recurrent dislocations may be serious with shoulder function being further compromised by every dislocation. Hence, patients are frequently limited in sports-related and social activities affecting quality of life (QoL) [11–14].

Treatment-wise, systematic reviews advocate for initial stabilising surgery in young, highly athletic, active, male patients with a primary TASD [1, 15] in spite of the expressed concern that early surgery exposes patients to unnecessary surgery-related complications and adds to societal treatment costs [16]. For other TASD patients (e.g. those aged 25–40 years, nonprofessional athletes, primary or recurrent dislocations) the evidence for optimal treatment (operative as well as nonoperative) is limited [1, 17]. Traditionally, post-traumatic standard care consists of closed reduction, followed by immobilisation in a shoulder sling and, if provided, some kind of physiotherapy [18]. Currently, there is no evidence-based exercise programme to prescribe for patients with TASDs [8] and the quantity and quality of studies investigating nonoperative treatment for this patient group is low [19]. Three randomised controlled trials (RCTs) investigating the effect of nonoperative (shoulder rehabilitation) versus operative treatment (arthroscopic or open Bankart procedures in addition to shoulder rehabilitation) in patients with primary TASD show that early operative reconstruction of the anterior capsulolabral complex is superior to nonoperative treatment in reducing redislocation rates [20–22]. However, the shoulder rehabilitation programmes used in these RCTs are poorly described making it difficult to reproduce, and they consist mostly of postoperative exercise principles. Thus, the nonoperatively treated patients may have been undertreated with specialised, intensive, nonoperative exercise regimes lacking [23]. Also, varying methodological issues exist that are expected to bias the findings of the RCTs [19, 23].

In other body regions, neuromuscular exercise has shown great potential in reducing joint pain, besides improving functional capacities and QoL [24–27]; e.g. neuromuscular exercise has shown to be as equally effective as early surgical reconstruction in patients with traumatic anterior cruciate ligament injuries in the knee, which is comparable with TASD on injury mechanism (traumatic origin, high impact), age (late teens to mid-30s), post-traumatic symptoms (pain, instability, loss of mechanical stability), besides reduction in physical and social function [24]. Hence, neuromuscular exercise also seems evident for patients with TASD due to loss of mechanical stability [28], and potentially impaired proprioceptive functions [29–31]. To our knowledge, such neuromuscular exercise programmes for increasing sensorimotor control and compensatory functional stability [25] have not yet been developed and tested scientifically on the shoulder [32].

In conclusion, the evidence for optimal treatment of patients with TASD (primary and recurrent) is lacking, and previous exercise programmes are poorly described and do not include the newest physiological knowledge for improving joint instability deficits. Sufficiently powered, good-quality and well-reported RCTs are needed to investigate nonoperative rehabilitation strategies in patients with TASD [33].

### Study objectives

The aim of this randomised controlled clinical trial is to evaluate the efficacy and safety of a 12-week, structured ‘Shoulder Instability Neuromuscular Exercise’ (SINEX) programme versus 12 weeks of a self-managed, training-based, shoulder exercise programme (‘standard care’), measured on the Western Ontario Shoulder Instability Index (WOSI) questionnaire [34] in patients with TASD (primary and recurrent). It is hypothesised that patients receiving 12 weeks of SINEX training will show superior improvement in short- and long-term shoulder-related QoL and function.

## Methods/design

### Study design

This trial is a multicentre research project in cooperation with three orthopaedic shoulder units in The Region of Southern and Northern Denmark. The study is a randomised (1:1), assessor-blinded, controlled trial with a parallel-group design investigating the efficacy and safety of a ‘SINEX training programme’ versus standard care for patients with TASD (primary and recurrent) with a primary endpoint after 12 weeks (3 months). The primary endpoint of 12 weeks was chosen since the primary effect measure is a patient-reported shoulder-related QoL outcome and not, as in previous trials, whether the shoulder dislocates or not. Thus, if there is no significant short-term effects of the SINEX programme, it is highly unlikely that there will be any long-term effects. Furthermore, long-term follow-up will be performed for all patients between 1 and 2 years from baseline. The protocol conforms to the recommendations from the EQUATOR network [35], using the Standard Protocol Items: Recommendations for Interventional Trials (SPIRIT) Checklist and Consolidated Standards of Reporting Trials (CONSORT) guidelines for RCTs [36, 37] Additional file 1.

### Settings and locations

Patients are recruited from shoulder outpatient clinics of orthopaedic departments in the Region of Northern Denmark (Aalborg University Hospital, Farsoe) and Southern Denmark (South-West Jutland Hospital, Esbjerg and Odense University Hospital, Odense).

### Participants

Participants included are classified as dynamic shoulder instability types (class B2) according to Gerber et al. [38], referring to patients with unidirectional shoulder instability initiated by trauma. Both men and women with primary or recurrent anterior shoulder dislocations are included, provided that they fulfil the following criteria:

#### Inclusion criteria

1. Age between 18 and 39 years
2. Minimum one radiographically verified anterior shoulder dislocation (total dissociation of the humeral head relative to the glenoid)
3. Self-reported shoulder trouble (physical and/or emotional), meaning diminished ability to participate or perform shoulder movements needed in sports/leisure activity and/or work tasks within the latest week

#### Exclusion criteria (any of the following)

1. Humeral fracture and/or bony Bankart lesion (visible on conventional radiographs at the time of presentation) warranted for surgery as decided by the orthopaedic surgeon
2. Pending surgery to the affected shoulder joint
3. More than five anterior shoulder dislocations (verified by patient record or subjective evaluation)
4. Suspected competing diagnosis (e.g. rheumatoid arthritis, cancer, neurological disorders, fibromyalgia, psychiatric diseases)
5. Sensory and motor deficits in the neck and/or shoulder
6. Pregnancy
7. Inadequacy in written and spoken Danish
8. Not willing or able to attend 12 weeks of supervised SINEX therapy

### Procedure

Patients consulting emergency departments with a TASD will be treated according to standard procedures consisting of shoulder reduction followed by sling immobilisation in internal rotation and use of analgesics, if needed. After completion of the subacute phase (3–6 weeks) patients will attend one of the participating shoulder units for a routine check of the actual shoulder function. Potential candidates are then identified and screened for initial eligibility by an orthopaedic surgeon according to inclusion criteria 1–2 and exclusion criteria 1–5. Patients fulfilling the eligibility criteria are referred to the principal investigator (first author), who performs the final eligibility assessment according to inclusion criterion 3 and exclusion criteria 5–8. All patients are provided with written and verbal information about the study objectives, and a signed consent is obtained for those eligible and willing to participate. Patients not included or declining to participate will be asked to fill out the WOSI questionnaire, and report age and gender so as to improve the selection bias analysis.

### Randomisation and concealment of allocation

Patients are randomly assigned to either of the two groups. An independent biostatistician, with no involvement in the clinical care of the patients, prepares a computer-generated list of random numbers (1:1), which is subsequently packed at each trial site into sequentially numbered, opaque, concealed envelopes, stating which group each individual is allocated to. Patients are stratified based on injury status: (1) primary (first time) or (2) recurrent (second to fifth) dislocations.

**Fig. 1:**
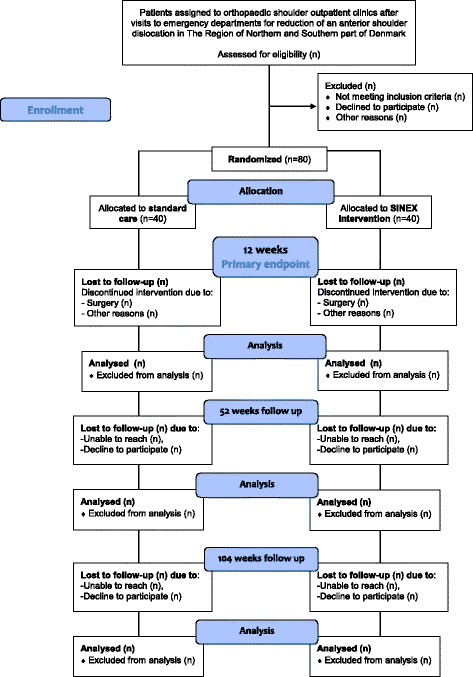
Flowchart of the Shoulder Instability Neuromuscular Exercise (SINEX) study

At each recruitment site, envelopes are stored in a closed room and managed by a single research assistant. After randomisation the same research assistant forwards group assignments to the treating physiotherapist who contacts the patient for an appointment of the first exercise instruction. Patients are reassessed after 3 months (12 weeks of exercise) and again after 12 (52 weeks) and 24 months (104 weeks). A flowchart of participants and randomisation is presented in Fig. 1.

### Blinding

The primary investigator and one research assistant, performing outcome measurements, are blinded according to treatment allocation and not involved in the treatment of patients. Though, blinding of treatment allocation for patient and physiotherapist is not possible. Patients involved are thoroughly informed (written and orally) that the optimal choice of treatment is truly unknown, thereby keeping the study hypothesis secret [36]. Further, to retain the blinding of the outcome assessors, patients are encouraged not to reveal their treatment assignment. Finally, all of the statistical analyses will be performed blinded according to group allocation and results will be interpreted in an author consensus statement prior to disclosing/revealing group allocation.

### Interventions

Both groups receive 12 weeks of active exercise treatment, information on correct ergonomic postures and instructions in active range of motion exercises and/or stretching of shoulder muscles if needed. Patients are asked not to seek other treatment for their current shoulder problem during the intervention period. After the end of intervention patients will be advised to continue their exercises and allowed to seek other treatment options. Patients experiencing additional dislocations or worsening of shoulder symptoms during the intervention period will be referred to an orthopaedic surgeon for a clinical evaluation of whether they can continue in the study. Withdrawn patients, due to either worsening of symptoms, surgery and/or retraction of participation consent, will be asked to complete all follow-up measurements to ensure a full dataset. Finally, patients are asked to fill out an exercise diary in relation to their home-based exercises.

### Control group

Participants allocated to standard care will begin their 12 weeks of self-managed training-based shoulder exercise programme with one introductory supervised physiotherapy session. Standard care consists of active exercises for the rotator cuff and scapular muscles as follows: strengthening exercises for the rotator cuff muscles (shoulder internal and external rotation with the subject’s elbow flexed to 90° and the elbow positioned at the subject’s side in addition to shoulder abduction performed inthe scapular plane. Mobility/co-activation of the scapular and core stability muscles is performed through the weight-bearing, four-point kneeling exercise, with simultaneous lift-off of one arm and the opposite leg. Patients are asked to perform the exercises three times weekly with 10 × 2 repetitions for each exercise. Participants are initially provided with a leaflet containing photos and descriptions of each exercise, besides general information about their current shoulder injury, potential future implications, and how to avoid pain and instability provoking shoulder movements. Finally, participants receive one phone call from a physiotherapist after 6 weeks of training to ensure progression and compliance with the exercises, in addition to having any patient-initiated communication that the patient may want to have regarding further shoulder-related questions. Patients allocated to standard care will be regarded as having completed the intervention with a minimum of two thirds (66%) of the planned home training.

### Intervention group

Participants allocated to the SINEX programme receive 12 weeks of individually physiotherapist-supervised exercise specifically, targeting the glenohumeral and scapular muscles. Moreover, functional kinetic chain exercises are incorporated for progressing to more difficult levels, mimicking daily activities and improving the transferability of everyday activities.

The SINEX programme is individually tailored within a standardised framework consisting of seven exercises (1–7): scapular setting and control (1), glenohumeral setting and control during internal (2) and external (3) rotation, co-contraction of glenohumeral muscles (4), dynamic glenohumeral stability (5), besides training of glenohumeral proprioception (6, 7).

Each exercise includes seven levels (A to G) of difficulty, ranging from a basic to an elite level. Exercises at the basic level (A to E) are performed with low load, large body support and focus on local shoulder muscle activity (quality before quantity). Exercises at the elite level (F to G) are performed with high load, less body support and increasing movement speed, according to the individual capability of each patient. The load and repetitions of each exercise are performed as follows; exercises 1–4 are performed according to the principle of strength training, meaning that basic and elite levels refer to low- and high-load training equal to 2 × 20–25 and 2 × 8–12 of 1 repetition maximum, respectively. Exercise 5 (dynamic shoulder stability) is performed with the use of time intervals equal to 3 × 10 and 3 × 20 seconds at basic and elite levels, respectively. For exercises 6 and 7 (proprioceptive training) the basic and elite levels are performed with 2 × 5 and 2 × 10 repetitions, respectively. Participants are instructed to perform each exercise at home as follows; exercises at basic levels (A to E) 7 days a week and exercises at elite levels (F to G) three times a week.

Participants are provided with online access to instructions and video recordings of each exercise and the accompanying levels of progression through the physiotherapy site www.digifys.com.

Participants are encouraged to progress exercises themselves, where relevant, with physiotherapists then evaluating the performance quality of each exercise at the supervised sessions. Hence, supervised sessions are provided throughout the 12 weeks, lasting approximately 45 min each. Supervised sessions are given twice a week for the first 2 weeks and then once a week for the remaining 10 weeks. Physiotherapists decide the amount of supervision, based upon movement control and capabilities of the individual patient. Participants allocated to SINEX are considered to have satisfactory completion of the supervised sessions with attendance of at least seven supervised sessions (out of 14 possible sessions) equal to 50% attendance. Furthermore, to be considered compliant with the intervention participants must have completed at least two thirds (66%) of the planned home-based exercises. For quality assurance, physiotherapists are instructed, and continuously encouraged, to attend at least seven supervised sessions within the 12-week intervention period. A full description of the exercise programme, including structure, content, progression guidelines and overall concept is provided in Additional file 2.

### Physiotherapists

To be involved as a physiotherapist, attendance at two training sessions on how to administer the individualised supervision of patients according to the two intervention protocols is required. Furthermore, all physiotherapists are initially provided with a ‘pilot’ patient to practice the individualised supervised sessions and must have completed at least 2 weeks of supervised sessions before starting up study participants. Physiotherapists are allowed to contact the primary investigator regarding any exercise-based challenges that they may have, though, without revealing treatment allocation of the individual participants.

### Data collection and follow-up

Two outcome assessors (primary investigator and one research assistant) perform all baseline and follow-up assessments. Before and during data collection outcome assessors train together the test procedures of objective outcomes to unify performance and interpretation. Also, thoroughly described test protocols for the objective outcome measurements are prepared.

### Baseline data

Table 1 describes the type of data and variables that are collected at various stages of the project. Furthermore, the following demographic and descriptive data are collected: gender, age, height (cm), weight (kg), injury mechanism (fall on the arm, pull in the arm, external force to the shoulder, other reasons), total number of dislocations and closed reductions at emergency units, dominant arm (left/right), injured shoulder (left/right), previous treatment due to shoulder symptoms? (no/yes; which treatment?), physical activity? (no/yes; h/week), educational level and employment status; medicine consumption? (no/yes; which?). Furthermore, to evaluate ‘equipoise’ [39], patients are asked to register their belief in the effect of the assigned treatment, shortly after randomisation, in relation to pain, function and QoL.

**Table 1:** Outcome measures to be collected

Outcome measure(s)	Data collection instrument	Time line for data collection
Primary		
WOSI _(total score)_	Mean score of 21 items (0–2100; 0 = no trouble)	0, 4, 8, 12, 52 and 104 weeks
Secondary (key secondary outcomes)		
Physical symptoms, Sport/recreation/work, lifestyle and emotions	Individual domains in the WOSI	0, 12, 52 and 104 weeks
Fear of movement and reinjury	Tampa Scale of Kinesiophobia	0, 12, 52 and 104 weeks
General health	EQ-5D-5 L	0, 12, 52 and 104 weeks
Pain intensities	Numeric Pain Rating Scales	0, 12, 52 and 104 weeks
Self-reported shoulder function	Patient Specific Functioning Scale	0 and 12 weeks
Self-reported and objective shoulder function	Constant-Murley Shoulder Score	0 and 12 weeks
Clinical shoulder instability	Clinical tests: apprehension, relocation and surprise	0 and 12 weeks
Shoulder proprioception (open chain)	Shoulder joint repositioning with use of laser pointer method	0 and 12 weeks
Shoulder proprioception (closed chain)	Nintendo Wii balance board	0 and 12 weeks
Maximum isometric shoulder muscle strength in 90° of abduction	Isoforce dynamometer	0 and 12 weeks
Demographic data and other measurements		
Total number of dislocations/subluxations	Questionnaire	0 and 12 weeks
Medication use	Questionnaire	12 weeks
Treatment satisfaction	Questionnaire	12 weeks
Patient-reported impression of treatment success	7-point Likert scale (GPE) (ranging from 1 = very much worse to 7 = very much improved)	Throughout
Adverse events	Physiotherapist records and questionnaire	Throughout

*WOSI* Western Ontario Shoulder Instability Index; *EQ-5D-5 L* EuroQol 5 dimensions; *GPE* Global Perceived Effect

### Primary outcome measure

The primary outcome measure is change from baseline to 12 weeks in the total score of the patient-reported outcome measurement the WOSI. The WOSI covers 21 items, ranging from 0 to 2100 (0 = no trouble) [34] (Table 1). The WOSI has been translated and adapted for use in Denmark, according to international guidelines, before inclusion of patients [40]. The WOSI is a relevant outcome measure due to its capability of capturing changes in disease-specific aspects of QoL in shoulder instability (activity-related shoulder symptoms, such as the ‘feeling of slipping’ and ‘being unable to trust the shoulder’) [41, 42]. As such, the WOSI is generally recommended as a patient-reported outcome when evaluating treatment effects of shoulder instability [43].

### Key secondary outcomes

A number of secondary patient-reported outcome measures are obtained: the four individual domains of the WOSI covering: 10 items in ‘Physical symptoms’ (0–1000), 4 items in ‘Sport/recreation/work’ (0–400), ‘Lifestyle’ (0–400) and ‘Emotions’ (0–300), with the level of ‘no trouble’ equal to 0 accounting for all domains [34]. Also, fear of movement and reinjury is evaluated with the use of Tampa Scale of Kinesiophobia (TSK; 17–68, 68 = highest fear of movement and reinjury) [44, 45], in addition to pain intensity at rest, within the latest 24 h and 7 days, respectively, using a Numeric Pain Rating Scale (NPRS; 0–10 score, 10 = worst imaginable pain) [46], besides patient-rated important activity with the Pain Specific Function Scale (PSFS, 0–10 score, 10 = no problems in performing the activity) [47]. For cost-effective analysis the total score of the EQ-5D-5 L (−0.59 to 1 score, −0.59 = lowest health-related quality of life) is used, whereas QoL is measured with the EQ-5D Visual Analogue Scale (EQ-VAS; 0 to 100, 0 = lowest health-related quality of life) [48].

Secondary objective outcome measures obtained (Table 1) are the Constant-Murley Shoulder Score (CMS; 0–100 score, 100 = best possible shoulder function [49], as used for self-reported and objective shoulder function. For comparison to normative data, strength and range of motion measurements were also obtained for the noninjured shoulder according to the CMS protocol [49]. Additionally, participants are screened for generalised joint hypermobility (GJH) with the use of Beighton’s criteria tests and criteria (0–9 score, <4 = no GJH) [50]. Beighton’s criteria have shown to be reliable for measuring GJH [51]. Specific evaluation of anterior shoulder instability is performed by the clinical tests apprehension, relocation and surprise (positive? yes/no) [52–54]. Finally, proprioceptive shoulder function is tested with the upper extremity in an open kinetic chain. Briefly, the test is performed as follows: patients are blindfolded and asked to actively reproduce different shoulder angles within low ranges of shoulder flexion and abduction (low equal to 60° ± 10°) using a laser beam pointing at a target scale. This method has previously shown satisfactory reliability [55] (Table 1).

### Demographic data and other measurements

A number of other outcomes are measured at the 12-week, 1 and 2-year follow-ups: patient-reported numbers of visits to the general practitioner or secondary health care system during and after the end of intervention, number of shoulder dislocations/subluxations, number of days sick listed from work and sport attributed to the actual shoulder injury, besides the number of referrals to or completed shoulder surgical procedures. Further, patients are asked to answer the question: ‘If you had the choice, would you then consider to have stabilising shoulder surgery performed?’ Finally, a self-rated impression of recovery is measured using the Global Perceived Effect Scale (GPE, seven-point Likert scale ranging from 1 to 7, 1 = very much worse and 7 = very much improved). The GPE is evaluated with the following question: ‘Compared to when this treatment first started, how would you describe your shoulder problems this latest week?’. The answer ‘no change’ (corresponding to 3 on the seven-point Likert scale) is considered a neutral response (Table 1).

Registration of adverse events or harms is obtained as follows; patients allocated to standard care will receive a phone call from a physiotherapist after 6 weeks of exercise; patients allocated to SINEX are monitored throughout the 12 weeks of exercise by the supervising physiotherapists continuously registering adverse events or harms. In addition, all patients are asked to fill out a short standardised questionnaire to record any adverse effects or harms at the 12-week follow-up.

### Sample size and power considerations

This study was designed as an exploratory (superiority) trial with two groups (SINEX and standard care) using the WOSI questionnaire, as recommended in clinical trials of shoulder instability patients [19, 43, 56]. The power and sample size calculation is based on the differences in the WOSI change score between the two groups from baseline to the 12-week follow-up.

It is expected that the group allocated to SINEX will improve 250 points more than the group allocated to standard care based on the primary outcome the WOSI at endpoint after 12 weeks. With a mean baseline WOSI score expected to be 1100 points (range 0–2100, with 2100 as worst possible score) and a common standard deviation assumed to be 320 [57], a sample size of 36 participants per group is required to detect a statistical difference (significance level of 0.05, two-sided, with 90% power). To account for possible barriers, noncompliant patients and participants lost-to-follow-up, it was decided to enrol a total sample size of 80 participants (40:40). For practical logistical reasons, new patients will no longer be included in the study after March 2017.

### Statistical evaluation

Primary analysis will be performed at the primary endpoint (12-week intervention period).

All descriptive statistics and tests will be reported in accordance with the recommendations of the ‘Enhancing the QUAlity and Transparency Of health Research’ (EQUATOR) network [35]; i.e. various forms of the CONSORT guidelines [58].

All primary analyses will follow the intention-to-treat principle; i.e. all participants in the trial will be included in the analysis according to the group to which they are randomised, regardless of dropout/any departures from allocated treatment. Missing data will be replaced using a nonresponder imputation, in which the baseline value is carried forward [59]. The rationale behind this type of analysis builds on the assumption that those who dropped out returned to their baseline WOSI score [60]. For sensitivity and exploratory purposes also a per-protocol analysis, including those with good compliance (as previously described) with the protocol (including outcome assessments available after 12 weeks) will be performed.

An analysis of covariance (ANCOVA) model will be used to analyse mean changes in continuous endpoints. The model will include treatment, study centre, and status with respect to type of dislocation as fixed effects, with the baseline value of the relevant variable as a covariate. Categorical outcomes for dichotomous endpoints will be analysed with the use of logistic regression with the same fixed effects and covariates as the respective ANCOVA.

For the longitudinal part of the trial a linear mixed ANCOVA model with repeated measures of the WOSI (4, 8 and 12 weeks) will be performed to test the difference over time between the intervention and the control group; interaction: Group × Time, with the same fixed effects and covariates as the respective ANCOVA. For these analyses the ‘data as observed’ will be applied (i.e. no imputation for missing data needed). An alpha level of 0.05 will be considered as being statistically significant (*p* < 0.05, two-sided). The data analysts will be blinded to the allocated interventions for primary analyses. In general, results will be expressed as the difference between group means with 95% confidence intervals and the associated *p* values.

Data analyses will be carried out according to a pre-established analysis plan, publicly available before the final patient is included, and analysed with the use of SAS software (SAS Institute Inc., Cary, NC, USA).

### Ethical considerations

Patients are informed about the randomised design with allocation to either of the two treatment arms and also about the content of the two treatment arms. Patients are kept blinded for any of the study hypotheses, which can be justified since patients are treated with either an anticipated equivalent, or superior, treatment to what they would have received if they did not participate in the study. Furthermore, both patient groups are expected to benefit from participation in the study through their respective interventions. The risk of sustaining another shoulder dislocation from participating in this study is not expected to be any higher than from regular daily activities. Furthermore, it is not anticipated that participation will cause any serious adverse events or harms. The trial will meet the criteria and principles of the Declaration of Helsinki (80) and has been approved by the local Ethics Committee for the Region of Southern Denmark (project ID: S-20140093). The trial is registered at ClinicalTrials.gov (ID; NCT02371928). Approval from The Danish Data Protection Agency is given (Journal number: 2015-57-0008)

## Discussion

Traumatic anterior shoulder dislocation is common and may decrease shoulder-related QoL. The aim of this randomised controlled clinical trial is to evaluate the efficacy and safety of a 12-week, structured, neuromuscular shoulder exercise programme versus 12 weeks of self-managed shoulder rehabilitation in patients with TASDs measured on the WOSI questionnaire.

The current study will shed light on the effect of nonoperative treatment for patients with primary and recurrent anterior shoulder dislocations and provide foundation for nonoperative treatment guidelines. Additionally, this trial will support evidence-based shared decision-making processes (between physicians and patients) in clinical practice, when patients seek orthopaedic and/or physiotherapy treatment for a primary or recurrent anteriorly dislocated shoulder.

Previously, only one level-II study has succeeded in managing TASD patients (average age 19 years) nonoperatively by including 12 weeks of specific shoulder rehabilitation exercise. At an average time to follow-up of 3 years 15 out of 20 patients (75%) had not redislocated their shoulder and had returned to sport within an average time of 3 months [61]. Thus, supervised and adequate progressive shoulder rehabilitation, therefore, seems evident following a TASD, but has not yet been documented in a high-level study such as in the current trial. Finally, no matter the treatment (operative as well as nonoperative), fear of movement and reinjury, mood, social support and self-motivation has shown to greatly influence the decision on return to sport [14]. Aspects like these are only covered by patient-reported outcomes, supporting the use of a patient-reported outcome as primary treatment effect measure in the current trial.

One limitation of the present trial may be that since little is known about the nonoperative treatment potential for patients with recurrent shoulder dislocation, the pragmatic choice of including patients with up to a maximum number of five dislocations may be too broad; although, orthopaedic surgeons will screen each patient before enrolment to make sure that no patients with injuries warranted for shoulder surgery are included. Furthermore, to secure an even distribution of primary and recurrent dislocation patients in each treatment group stratification techniques are used. Finally, as in similar exercise trials, it is not possible to blind either patients or physiotherapists to the treatment allocation. Thus, only the testers can be blinded.

The study strengths are the rigorous inclusion criteria compared with previous trials of TASDs, aiming at a homogenous patient group with self-reported shoulder trouble. Due to the fact that not all patients experience post-traumatic shoulder trouble nor recurrent shoulder instability, unnecessary invasive procedures will not initially take place in the current study, as compared to previous RCTs performing immediate stabilising surgery [20–22]. Finally, the use of a standardised, individualised and physiotherapist-supervised neuromuscular shoulder exercise programme developed for accommodating all types of TASD patients (e.g. those with severe instability and pain and those in need of more sports-specific exercises), is an additional strength.

### Timeline, trial status and publication plan

Recruitment of patients began March 2015 and is expected to run consecutively until March 2017. At the time of submission of this study protocol (October 2016), the trial is ongoing and still recruiting patients. For the time being, 50 patients have been included in the project. Data will be analysed, interpreted and published regardless of whether results are positive, negative or inconclusive.

## Additional files

Additional file 1: SPIRIT 2013 Checklist: recommended items to address in a clinical trial protocol and related documents*. (DOC 121 kb)
Additional file 2: Shoulder Instability Neuromuscular Exercise (SINEX) program. Description of the concept, structure, and content of the SINEX program. (PDF 4494 kb)

## Abbreviations

ANCOVA: Analysis of covariance
QoL: Quality of life
RCT: Randomised controlled trial
SINEX: Shoulder Instability Neuromuscular Exercise
TASD: Traumatic anterior shoulder dislocation
WOSI: Western Ontario Shoulder Instability Index

## References

[1] Longo UG (2014). Management of primary acute anterior shoulder dislocation: systematic review and quantitative synthesis of the literature. Arthroscopy.

[2] Sofu H (2014). Recurrent anterior shoulder instability: review of the literature and current concepts. World J Clin Cases.

[3] Kroner K, Lind T, Jensen J (1989). The epidemiology of shoulder dislocations. Arch Orthop Trauma Surg.

[4] Zacchilli MA, Owens BD (2010). Epidemiology of shoulder dislocations presenting to emergency departments in the United States. J Bone Joint Surg Am.

[5] Romeo AA, Cohen BS, Carreira DS (2001). Traumatic anterior shoulder instability. Orthop Clin North Am.

[6] Liavaag S (2011). The epidemiology of shoulder dislocations in Oslo. Scand J Med Sci Sports.

[7] Leroux T (2014). Epidemiology of primary anterior shoulder dislocation requiring closed reduction in Ontario, Canada. Am J Sports Med.

[8] Monk AP (2015). Evidence in managing traumatic anterior shoulder instability: a scoping review. Br J Sports Med.

[9] Simonet WT, Cofield RH (1984). Prognosis in anterior shoulder dislocation. Am J Sports Med.

[10] Olds M (2015). Risk factors which predispose first-time traumatic anterior shoulder dislocations to recurrent instability in adults: a systematic review and meta-analysis. Br J Sports Med.

[11] Dawson J, Fitzpatrick R, Carr A (1996). Questionnaire on the perceptions of patients about shoulder surgery. J Bone Joint Surg (Br).

[12] Matsen FA, Zuckerman JD (1983). Anterior glenohumeral instability. Clin Sports Med.

[13] Gartsman GM (1998). Self-assessment of general health status in patients with five common shoulder conditions. J Shoulder Elbow Surg.

[14] Tjong VK (2015). A qualitative investigation of return to sport after arthroscopic Bankart repair: beyond stability. Am J Sports Med.

[15] Handoll HH, Almaiyah MA, Rangan A (2004). Surgical versus non-surgical treatment for acute anterior shoulder dislocation. Cochrane Database Syst Rev.

[16] Sachs RA (2007). Can the need for future surgery for acute traumatic anterior shoulder dislocation be predicted?. J Bone Joint Surg Am.

[17] Handoll HH (2006). Conservative management following closed reduction of traumatic anterior dislocation of the shoulder. Cochrane Database Syst Rev.

[18] O’Brien SJ, Warren RF, Schwartz E (1987). Anterior shoulder instability. Orthop Clin North Am.

[19] Gibson K (2004). The effectiveness of rehabilitation for nonoperative management of shoulder instability: a systematic review. J Hand Ther.

[20] Bottoni CR (2002). A prospective, randomized evaluation of arthroscopic stabilization versus nonoperative treatment in patients with acute, traumatic, first-time shoulder dislocations. Am J Sports Med.

[21] Jakobsen BW (2007). Primary repair versus conservative treatment of first-time traumatic anterior dislocation of the shoulder: a randomized study with 10-year follow-up. Arthroscopy.

[22] Kirkley A (1999). Prospective randomized clinical trial comparing the effectiveness of immediate arthroscopic stabilization versus immobilization and rehabilitation in first traumatic anterior dislocations of the shoulder. Arthroscopy.

[23] Yuen CK, To DB (2008). Is operative repair better than conservative treatment after primary anterior shoulder dislocation?. Arthroscopy.

[24] Frobell RB (2010). A randomized trial of treatment for acute anterior cruciate ligament tears. N Engl J Med.

[25] Ageberg E, Link A, Roos EM (2010). Feasibility of neuromuscular training in patients with severe hip or knee OA: the individualized goal-based NEMEX-TJR training program. BMC Musculoskelet Disord.

[26] Stensrud S, Roos EM, Risberg MA (2012). A 12-week exercise therapy program in middle-aged patients with degenerative meniscus tears: a case series with 1-year follow-up. J Orthop Sports Phys Ther.

[27] Eitzen I (2010). A progressive 5-week exercise therapy program leads to significant improvement in knee function early after anterior cruciate ligament injury. J Orthop Sports Phys Ther.

[28] Hayes K (2002). Shoulder instability: management and rehabilitation. J Orthop Sports Phys Ther.

[29] Smith RL, Brunolli J (1989). Shoulder kinesthesia after anterior glenohumeral joint dislocation. Phys Ther.

[30] Lephart SM (1994). Proprioception of the shoulder joint in healthy, unstable, and surgically repaired shoulders. J Shoulder Elbow Surg.

[31] Edouard P, et al. "Sensorimotor control deficiency in recurrent anterior shoulder instability assessed with a stabilometric force platform." J Shoulder Elbow Surg. 2014;23(3):355-60. 10.1016/j.jse.2013.06.005 24007652

[32] Zech A (2009). Neuromuscular training for rehabilitation of sports injuries: a systematic review. Med Sci Sports Exerc.

[33] Hanchard NC, Goodchild LM, Kottam L (2014). Conservative management following closed reduction of traumatic anterior dislocation of the shoulder. Cochrane Database Syst Rev.

[34] Kirkley A (1998). The development and evaluation of a disease-specific quality of life measurement tool for shoulder instability. The Western Ontario Shoulder Instability Index (WOSI). Am J Sports Med.

[35] Christensen R, Bliddal H, Henriksen M (2013). Enhancing the reporting and transparency of rheumatology research: a guide to reporting guidelines. Arthritis Res Ther.

[36] Chan AW (2013). SPIRIT 2013 explanation and elaboration: guidance for protocols of clinical trials. BMJ.

[37] Schulz KF (2010). CONSORT 2010 statement: updated guidelines for reporting parallel group randomised trials. PLoS Med.

[38] Gerber C, Nyffeler RW (2002). Classification of glenohumeral joint instability. Clin Orthop Relat Res.

[39] Freedman B (1987). Equipoise and the ethics of clinical research. N Engl J Med.

[40] Guillemin F, Bombardier C,Beaton D. "Cross-cultural adaptation of health-related quality of life measures: literature review and proposed guidelines." J Clin Epidemiol. 1993;46(12):1417-1432. 10.1016/0895-4356(93)90142-n 8263569

[41] Salomonsson B (2009). The Western Ontario Shoulder Instability Index (WOSI): validity, reliability, and responsiveness retested with a Swedish translation. Acta Orthop.

[42] Hofstaetter JG (2010). Cross-cultural adaptation and validation of the German version of the Western Ontario Shoulder Instability index. Arch Orthop Trauma Surg.

[43] Plancher KD, Lipnick SL (2009). Analysis of evidence-based medicine for shoulder instability. Arthroscopy.

[44] Vlaeyen JW (1995). Fear of movement/(re)injury in chronic low back pain and its relation to behavioral performance. Pain.

[45] Mintken PE (2010). Psychometric properties of the Fear-Avoidance Beliefs Questionnaire and Tampa Scale of Kinesiophobia in patients with shoulder pain. Arch Phys Med Rehabil.

[46] Downie WW (1978). Studies with pain rating scales. Ann Rheum Dis.

[47] Donnelly C, Carswell A (2002). Individualized outcome measures: a review of the literature. Can J Occup Ther.

[48] Rabin R, de Charro F (2001). EQ-5D: a measure of health status from the EuroQol Group. Ann Med.

[49] Ban I (2013). Standardised test protocol (Constant Score) for evaluation of functionality in patients with shoulder disorders. Dan Med J.

[50] Beighton P, Solomon L, Soskolne CL (1973). Articular mobility in an African population. Ann Rheum Dis.

[51] Juul-Kristensen B (2007). Inter-examiner reproducibility of tests and criteria for generalized joint hypermobility and benign joint hypermobility syndrome. Rheumatology (Oxford).

[52] Lo IK (2004). An evaluation of the apprehension, relocation, and surprise tests for anterior shoulder instability. Am J Sports Med.

[53] Vind M (2011). Inter-examiner reproducibility of clinical tests and criteria used to identify subacromial impingement syndrome. BMJ Open.

[54] Tzannes A (2004). An assessment of the interexaminer reliability of tests for shoulder instability. J Shoulder Elbow Surg.

[55] Vafadar AK, Cote JN, Archambault PS. Interrater and intrarater reliability and validity of 3 measurement methods for shoulder-position sense. J Sport Rehabil. 2016;Technical Report 19:2014–0309. 10.1123/jsr.2014-0309 25962861

[56] Robinson CM (2006). Functional outcome and risk of recurrent instability after primary traumatic anterior shoulder dislocation in young patients. J Bone Joint Surg Am.

[57] Cacchio A (2012). Cross-cultural adaptation and measurement properties of an Italian version of the Western Ontario Shoulder Instability Index (WOSI). J Orthop Sports Phys Ther.

[58] Moher D (2010). CONSORT 2010 explanation and elaboration: updated guidelines for reporting parallel group randomised trials. BMJ.

[59] Little RJ (2012). The prevention and treatment of missing data in clinical trials. N Engl J Med.

[60] White IR (2011). Strategy for intention to treat analysis in randomised trials with missing outcome data. BMJ.

[61] Aronen JG, Regan K (1984). Decreasing the incidence of recurrence of first time anterior shoulder dislocations with rehabilitation. Am J Sports Med.

