# Supplementary material for: A neuromuscular exercise programme versus standard care for patients with traumatic anterior shoulder instability: study protocol for a randomised controlled trial (the SINEX study)
Source: Trials. 2017 Feb 28;18:90. doi: 10.1186/s13063-017-1830-x (PMC5331774; doi:10.1186/s13063-017-1830-x)
Supplement: Additional file 2: — Shoulder Instability Neuromuscular Exercise (SINEX) program. Description of the concept, structure, and content of the SINEX program. (PDF 4494 kb) [file 13063_2017_1830_MOESM2_ESM.pdf]

Additional file 1

Shoulder Instability Neuromuscular Exercise (SINEX) program

Developed by

Henrik Eshøj PT, MSc, PhD Fellow, Gorm Høi Jensen PT, MSc,

Karen Søgaard, Prof. & Birgit Juul-Kristensen PT, PhD

In the following, a description of the concept, structure, and content of a physical therapist-supervised, intensive and individualized progressed Shoulder Instability Neuromuscular Exercise (SINEX) program for treating patients with traumatic anterior shoulder dislocations (primary and recurrent) is provided. The SINEX program contains a varying number of exercises targeting the glenohumeral and scapular stabilizing muscles, to centralize the humeral head back into the glenoid.

The overall purpose of the SINEX program is to improve the sensorimotor control of the shoulder and achieve compensatory functional stability through increased muscle strength and restoration of kinetic muscle chain activity and core stability.

The program runs for 12 weeks with the options of a number of physical therapist-supervised sessions, lasting approximately 45 minutes each. A cornerstone in the SINEX program is movement quality. Hence, each patient is supervised individually and each exercise is adjusted and progressed according to the quality of movement during exercise performance.

The SINEX program consists of five focus areas (scapular and glenohumeral setting and control, shoulder muscle co-contraction, dynamic shoulder stability, besides shoulder proprioception), including seven exercises targeting specific anatomical structures and biomechanical functions.

Each exercise has seven levels of difficulty ranging from basic (A-E) to elite (F-G) levels, respectively. The aim of the exercises at basic level is to re-educate the neuromuscular system and to improve the functional shoulder stability. When accomplished, patients are progressing into elite level exercises, consisting of functional whole body exercises and "return to sport " movements to improve the transferability into every-day actions.

Development of the exercise program

The neuromuscular exercise program was developed based on literature, in which the post-traumatic pathophysiological consequences of having an instable shoulder were described [1-9]. Further, neuromuscular theories coupled with exercise experiences and clinical practice from similar musculoskeletal disorders [10-15], in addition to input from clinicians/physiotherapists with theoretical and practical knowledge of neuromuscular training aspects, formed the basis of the exercise program. Exercise strategies, frequencies and content of the program were discussed, rehearsed and adjusted on several occasions.

Patient-responsibility and self-management

The SINEX program is developed with special emphasis on patient-responsibility and self-management of rehabilitation. Hence, patients are provided with knowledge on how to act if symptoms of shoulder pain and instability re-occur during home exercise and activities of daily living. Initially, the physical therapist determines the levels of each exercise, based on quality assessment of the current shoulder function of each individual. As patients become more familiar

with the SINEX concept, (how to perform exercises with quality and when to progress, adapt and act in relation to their current shoulder function, etc), patients are emphasized and encouraged to progress to new levels of exercise by themselves, through a web-based platform (see below).

Web-based access to exercise instructions

To increase movement quality and compliance with the home exercises patients are provided with online access of video recordings and verbal instructions of each exercise and progression levels through the web-based exercise platform www.digifys.com.

Physical therapist-supervised sessions

The following elements are incorporated in the supervision of patients to increase compliance and educate patients to self-management of exercises. Patients are taught to differentiate between inflammatory symptoms (not acceptable during or after exercises) and muscle soreness, and how to act in relation to these symptoms (see Figure 1). Patients are informed about the relevance of each exercise, and taught when/how to correct their body posture and movement quality, according to prescriptions for satisfactory neuromuscular control.

At the first supervision patients are provided with a small leaflet introducing patients for two simple tools with the purpose of increasing the home exercise compliance, as follows:

-Management of shoulder symptoms during home exercises

Management of shoulder pain and/or instability during exercises is performed with the use of a symptom-scale depicted below (Figure 1) ranging from 0-10, with 10 being worst imaginable symptoms. Exercises performed with symptoms exceeding five (red area) need adjustments to avoid worsening of symptoms.

Figure 1.

Symptom scale

No symptoms	0	1	2	3	4	5	6	7	8	9	10	Worst imaginable symptoms
		Safe			Acceptable				High risk			

-Management of load during home exercises

To ensure optimal exercise load during exercises a strength-scale, as depicted below (Figure 2), is used. When performing the exercises at basic (A-E) and elite levels (F-G), the resistance should feel light to moderate (green-yellow area) and moderate to heavy (yellow-red area), respectively.

Figure 2.
Strength scale

Light	Moderate	Heavy

Progression of exercises

To allow for progression each exercise has seven levels of difficulty (five basic and two elite levels, respectively). Exercises are progressed when satisfactory neuromuscular control is obtained, according to the following criteria; exercise performed with movement quality, resistance and repetition accomplished, no compensating movement strategies, performed within the accepted limit of symptoms (symptom scale, Figure 1), steady breathing and general body control, no need for visual, verbal or tactile feedback.

Below, the SINEX progam at a glance (Table 1) is provided.

Online material for Eshoj et al.
A neuromuscular exercise program for patients with traumatic anterior shoulder instability

Table 1.
Concept, structure and content of the SINEX program

**Exercise focus**	1) Scapula setting and control<br>2) Glenohumeral setting and control (during internal glenohumeral rotation)<br>3) Glenohumeral setting and control (during external glenohumeral rotation)<br>4) Glenohumeral muscle co-contraction<br>5) Dynamic glenohumeral muscle stability<br>6) Glenohumeral proprioception (use of an exercise ball)<br>7) Glenohumeral proprioception (use of a laser pointer)
**Determination of exercise level**	- Physical therapist demonstrates the exercise<br>- Patient performs the exercise<br>- Physical therapist evaluates exercise performance and quality until an appropriate level is found
**Criteria for progression**	Satisfactory neuromuscular control (which determines progression of exercises) is defined as follows:<br><br>- Exercise performed as described<br>- Load and repetitions achieved<br>- Symptoms < 5 (on the pain and instability symptom-scale, ranging from 0-10, 10 being worst)<br>- No need for visual, verbal or tactile feedback (from physical therapist, mirrors, etc.)<br>- Movement quality throughout (No compensating strategies)
**Exercise adjustments**	If criteria for progression are accomplished at one exercise level, but the next level causes symptoms >5 on the pain and instability symptom-scale, one of two options may be used:<br><br>**-1)** Patient continues at the exercise level where criteria for progression is obtained, but is challenged by minimizing base of body support and/or exercise with eyes closed<br><br>**-2)** Patient progress to the exercise level that could not be performed according to symptoms, but the exercise is adjusted so that it can be accomplished according to the pain and instability symptom-scale and with satisfactory movement quality.<br><br>For further progression, any adjusted exercises must be performed as originally described with satisfactory neuromuscular control
**Exercise difficulty**	
- Basic (A-E)	Low load (2x20-25 repetition-maximum (RM))<br>Large base of body support<br>Focus on local muscular activity (quality before quantity)<br>Movement speed during exercise equal to counts of 1-2-3-3-2-1
- Elite (F-G)	High load (2x8-12 RM)<br>Minimal base of body support<br>Focus on local and global muscular activity (core, functional movements, muscle chains)<br>Movement speed during exercise adjusted to individual capabilities
**Exercise frequency**	
- Basic level (A-E)	Every day
- Elite level (F-G)	Three times a week
**Supervised sessions**	1-2 supervisions per week in the first two weeks<br>1 supervision per week for the remaining ten weeks<br><br>The amount of supervisions will be based upon individual capabilities and movement qualities during the supervised sessions
**General instruction and add-on´s**	Avoidance of slouched position and protracted shoulders in general<br>Shoulder range of motion exercises and/or stretching of neck muscles if needed

Specific exercise descriptions

Exercise 1 Scapular setting and control

Biomechanical purpose: To optimize the position and movement of the scapular

Generally: Avoid compensating strategies such as over-activation of the trapezius superior (excessive shoulder elevation) during the exercise

Purpose from a patient perspective: *"This exercise is important for the overall function of your shoulder, since the shoulder blades are, partly, responsible for a good shoulder function"*

Basic level (2x20-25 repetitions)

Level	Performance	Photos	Feedback to the patient
**A**	Prone lying, arms at side<br><br>Activate the middle and lower trapezius to set the scapula<br><br>Count to five and let go again	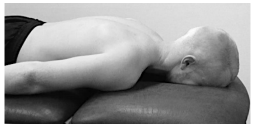 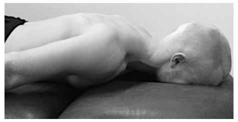	Facilitate the exercise by placing the fingers of the opposite hand on the back and inferior to the scapula.<br><br>Activate the scapular muscles and sense that the angulus inferior approaches the tip of the fingers of the opposite hand
**B**	Sitting erect on an exercise ball with both feet on the ground<br>Neck and lower back in neutral<br>Arms resting<br><br>Activate the middle and lower trapezius to set the scapula<br><br>Keep the scapular setting while turning the head slowly from side to side.	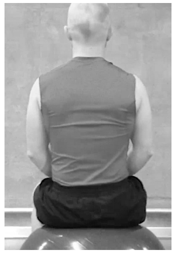 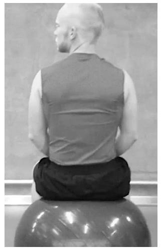	As above or with the opposite hand placed on front of the shoulder to sense that the shoulder is slightly retracted during scapular setting

**C**	Starting position as in B<br><br>Activate the middle and lower trapezius to set the scapula<br><br>Control the scapular setting while moving both arms towards the ceiling in the scapular plane, elbows extended and thumbs up<br><br>Lower arms again while controlling the scapula, relax and repeat	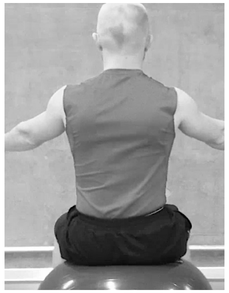 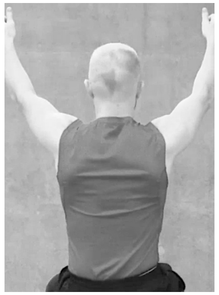	Use of mirror
**D**	Starting position as in C<br><br>Lift the leg of the injured side from the ground<br><br>With one leg lifted, perform the same exercise as in C<br><br>Lower the arms again while controlling the scapula, relax and repeat	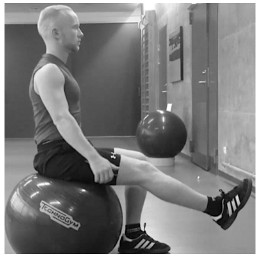 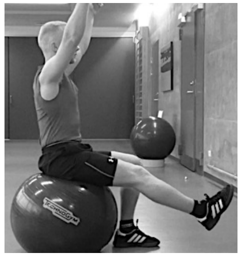	As above
**E**	Starting position as in D<br><br>Place one end of a theraband tube underneath the foot opposite to the injured shoulder and the other end in the hand at the injured side<br><br>Activate the middle and lower trapezius to set the scapula<br><br>Control the scapular setting while moving both arms towards the ceiling in the scapular plane, elbows extended and thumbs up<br><br>Lower the arms again while controlling the scapula, relax and repeat	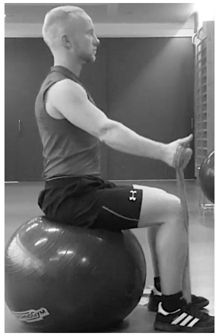 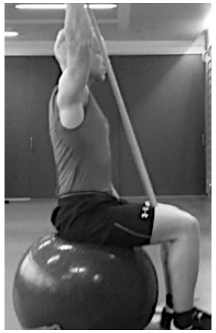	As above

Elite level (2x8-12 repetitions)			
**F**	Standing on both feet<br>Upper and lower back in neutral<br><br>Place one end of a theraband tube underneath the foot opposite to the injured shoulder and the other end in the hand of the injured shoulder<br>Activate the middle and lower trapezius to set the scapula and flex the injured shoulder with elbow extended and thumb pointing towards the ceiling as in E<br>With shoulder fully flexed, lower the arm slightly to approximately 150 degrees of shoulder flexion<br>Tighten the theraband tube by extending the other arm in front of the body<br>Move the injured arm above the head again while keeping the theraband tight<br><br>When fully flexed, release the tension of the theraband and lower the injured arm again, relax and repeat	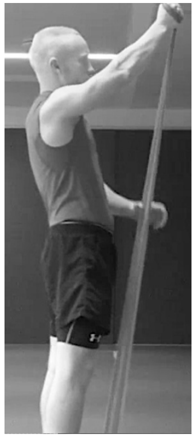 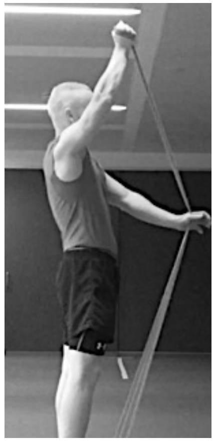	As above
**G**	As in F, but with the leg at the injured side lifted from the floor<br><br>If further progression is needed:<br>Increase the speed or the resistance of the exercise, close the eyes	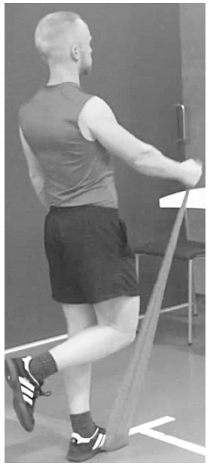 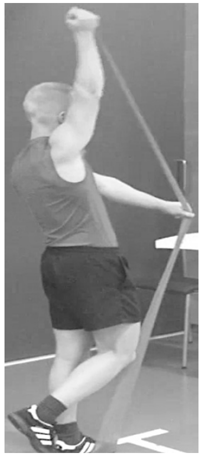	As above

Exercise 2	Glenohumeral setting and control (internal rotation)

**Biomechanical purpose:** To optimize the position and control of the glenohumeral joint during rotational movements.
**Generally:** Avoid compensating strategies during glenohumeral setting such as over-activation of scapular adductor muscles (excessive shoulder retraction)
**Purpose from a patient perspective:** *"This exercise is important for the overall function of your shoulder, since these muscles will help to position your shoulder joint correctly during movements. A poorly positioned shoulder joint often results in pain and discomfort during shoulder movements"*

Basic level (2x20-25 repetitions)			
**Level**	**Performance**	**Photos**	**Feedback to the patient**
**A**	Lying supine with knees flexed<br><br>Injured shoulder in 45 degrees of abduction, elbow flexed 90 degrees<br><br>Place a folded towel underneath the elbow to obtain neutral alignment between the elbow and shoulder<br><br>Activate the subscapularis to retract the glenohumeral head slightly into the cavitas glenoidalis<br><br>Count to five, relax and repeat	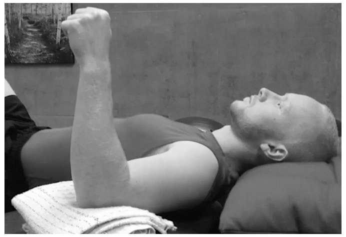	Facilitate the exercise by placing the opposite fingers in front of the injured shoulder<br><br>Sense that the shoulder moves away from the fingers<br><br>Alternatively, place a thin, folded towel on the back of the shoulder joint. To retract the glenohumeral head back into the cavitas, squize the towel slightly<br><br>Make sure that it is a local glenohumeral joint retraction (small movement) and not a retraction of the entire shoulder belt
**B**	Starting position as in A<br><br>Set the glenohumeral joint as in A<br><br>Rotate the glenohumeral joint into internal and external rotation while maintaning the glenohumeral setting<br><br>The axis of movement should be through the humerus	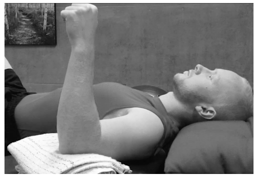 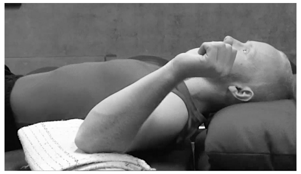	As above

**C**	Lying supine with knees flexed<br>Injured shoulder in 90 degrees of abduction, elbow flexed 90 degrees<br>Place a folded towel underneath the elbow to obtain neutral alignment between the elbow and shoulder<br><br>Set the glenohumeral joint<br><br>Rotate the glenohumeral joint into internal rotation with the use of a theraband as resistance (concentric activation)<br><br>Return to neutral while controlling and maintaining the glenohumeral setting (eccentric activation)<br><br>The axis of movement should be through the humerus	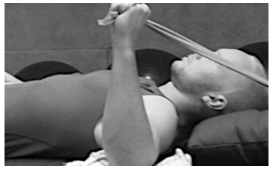 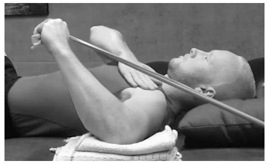	As above<br><br>Furthermore, a laser pointer, fixed at the wrist, pointing at an imaginary line at the ceiling may be used as visual feedback to control the movement (axis through the humerus)
**D**	Starting position as in C – but with no support underneath the elbow<br><br>Set the glenohumeral joint<br><br>Rotate the glenohumeral joint into internal rotation and back to neutral while controlling and maintaining the glenohumeral setting	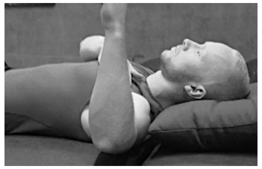 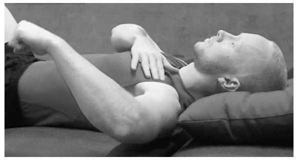	As above
**E**	Fix one end of a theraband behind the body just above shoulder height<br>Place the other end of the theraband in the hand at the injured side<br><br>Standing with the leg opposite to the injured shoulder in front, knees slightly flexed, bodyweight transferred to the front leg (front knee aligned with the hip and foot)<br>Upper and lower back positioned in neutral<br><br>Place the shoulder in 90 degrees of abduction, elbow flexed, shoulder in neutrol rotation (hand against the ceiling)<br><br>Set the glenohumeral joint<br><br>Rotate the glenohumeral joint into internal rotation with the use of a theraband as resistance (concentric activation)<br><br>Return to neutral while controlling and maintaining the glenohumeral setting (eccentric activation)	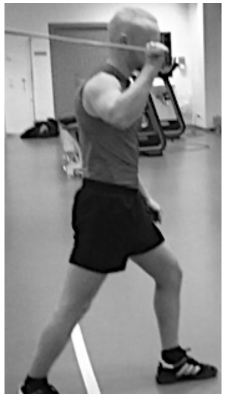 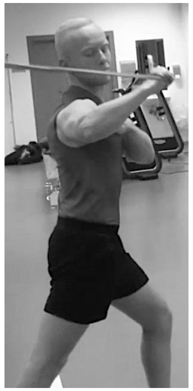	Furthermore, a laser pointer, fixed above the elbow, pointing at a fixed point can be used as visual feedback to control the movement (axis through the humerus)

Elite level (2x8-12 repetitions)			
**F**	As in E, but with the injured shoulder in 120-130 degrees of abduction (throwing position)	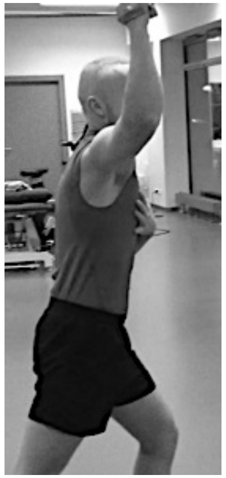 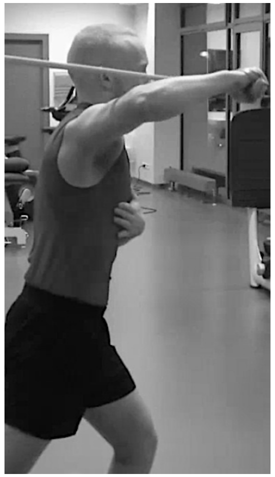	As above
**G**	As in F, but only with the leg opposite to the injured shoulder in the ground<br><br>If further progression is needed:<br>Increase the shoulder abduction angle, increase the speed or the resistance of the exercise, close the eyes	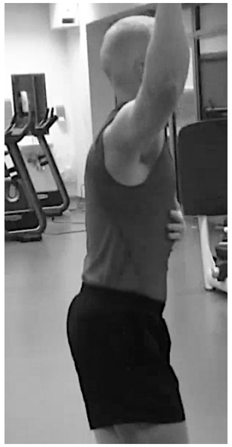 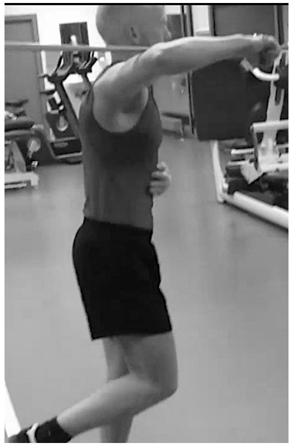	As above

Exercise 3 Glenohumeral setting and control (external rotation)

**Biomechanical purpose:** To optimize the position and control of the glenohumeral joint during rotational movements.
**Generally:** Avoid compensating strategies during glenohumeral setting such as over-activation of scapular adductor muscles (excessive shoulder retraction)
**Purpose from a patient perspective:** *"This exercise is important for the overall function of your shoulder, since these muscles will help to position your shoulder joint correctly during movements. A poorly positioned shoulder joint often results in pain and discomfort during shoulder movements"*

Basic level (2x20-25 repetitions)			
**Level**	**Performance**	**Photos**	**Feedback to the patient**
**A**	This exercise is the same as 2A and should only be performed once	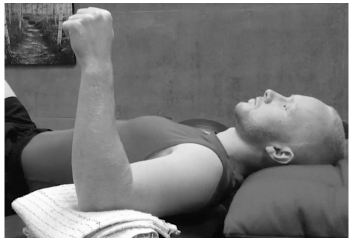	As in 2A
**B**	This exercise is the same as 2B and should only be performed once	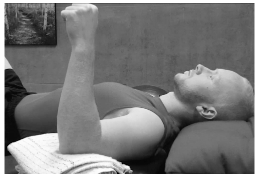 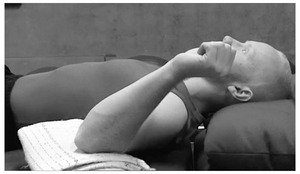	As in 2B

**C**	Starting position as in 2C<br><br>Set the glenohumeral joint<br><br>Rotate the glenohumeral joint into external rotation with the use of a theraband as resistance (concentric activation)<br><br>Return to neutral while controlling and maintaining the glenohumeral setting (eccentric activation)<br><br>The axis of movement should be through the humerus	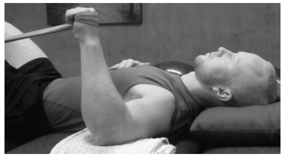 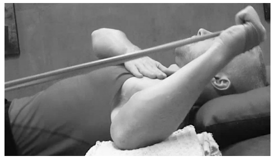	As in 3C
**D**	This exercise is the same as 2D and should only be performed once	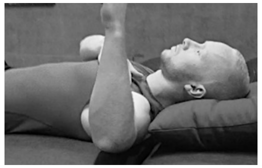 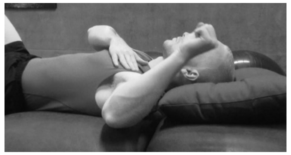	As in 2D
**E**	Fix one end of a theraband in front of the body in hip height<br><br>Place the other end of the theraband in the hand at the injured side<br><br>Starting position as in 2E<br><br>Place the shoulder in 90 degrees of abduction, elbow flexed, shoulder in 90 degrees of internal rotation (hand pointing forward)<br><br>Set the glenohumeral joint<br><br>Rotate the glenohumeral joint into external rotation with the use of a theraband as resistance (concentric activation)<br><br>Return to starting position while controlling and maintaining the glenohumeral setting (eccentric activation)	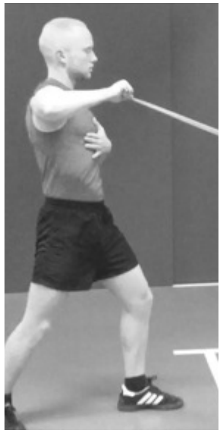 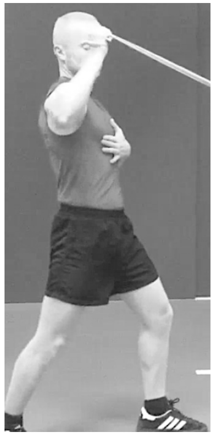	As in 2E

Elite level (2x8-12 repetitions)			
**F**	As in 3E, but with the injured shoulder in 120-130 degrees of abduction (throwing position)	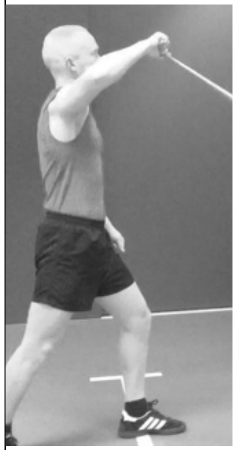 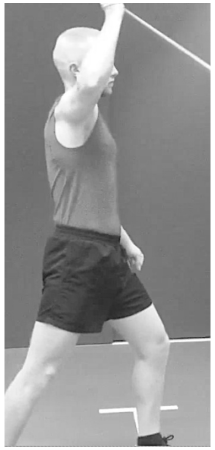	As in 2F
**G**	As in 3F, but only with the leg opposite to the injured shoulder in the ground<br><br>If further progression is needed:<br>Increase the shoulder abduction angle, increase the speed or the resistance of the exercise, close the eyes	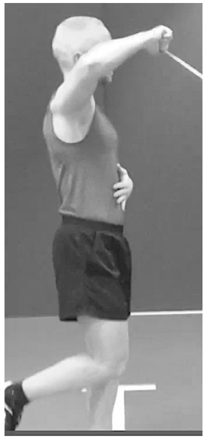 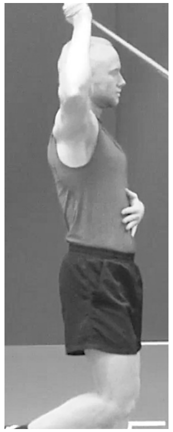	As in 2G

Exercise 4 Glenohumeral muscle co-contraction

**Biomechanical purpose:** To optimize the simultaneous activation of the shoulder agonist and antagonist muscles besides core muscle training and activation of the shoulder proprioceptive system.
**Purpose from a patient perspective:** *”This exercise is important to improve your ability to stabilize and activate the muscles surrounding your shoulder simultanoesuly. This is important to manage all of your daily shoulder movements”*

Basic level (2x20-25 repetitions)

Level	Performance	Photos	Feedback to the patient
**A**	Prone lying with an exercise ball supporting the lower extremities (underneath the thighs) and arms extended carrying the weight of the upper body.<br><br>Activate the scapular muscles and protract the shoulder girdle performing a push-up plus<br><br>Roll backwards to flex the shoulders, relax and repeat	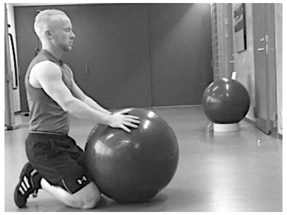 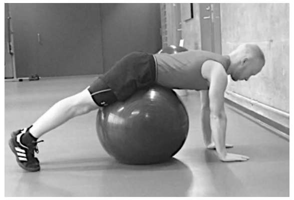	Sence that your shoulder blades move forward on the chest
**B**	Starting position as in A<br><br>Activate the scapular muscles and protract the shoulder girdle performing a push-up plus<br><br>Shift the weight from side to side<br><br>Avoid scapular winging	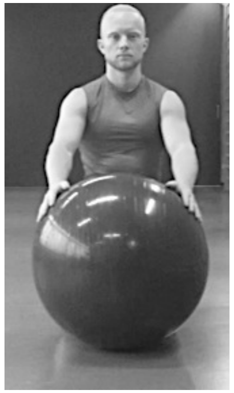 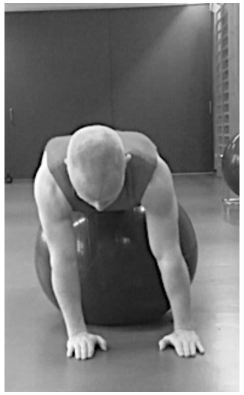	Sence that your shoulder blades move forward on the chest

**C**	Standing with feets in hip distance apart. Lean forward against an exercise ball placed in chest height at a corner<br><br>Activate the scapular muscles and protract the shoulder girdle performing a push-up plus while lifting one arm at a time<br><br>Avoid scapular winging	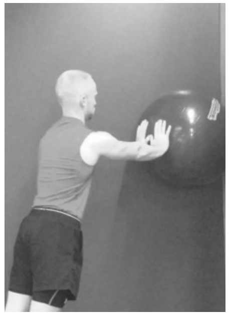 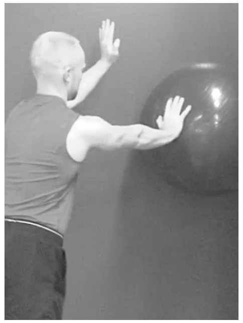	Sence that your shoulder blades move forward on the chest and keep this position
**D**	Prone lying with an exercise ball supporting the lower extremities (underneath the thighs)<br><br>Both arms extended carrying the weight of the upper body.<br><br>Activate the scapular muscles and protract the shoulder girdle performing a push-up plus<br><br>“Walk” forward on both arms while keeping control of the scapula<br><br>Roll back, repeat	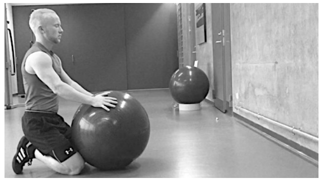 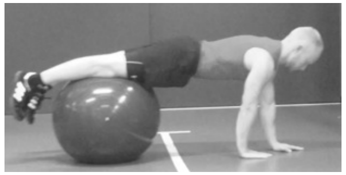	As in 4C
**E**	Starting position as in 4D<br><br>“Walk” forward on both arms<br><br>Flex both elbows, followed by a push-up plus (keep lower back in neutral)<br><br>Roll back, repeat	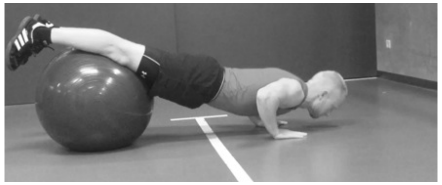 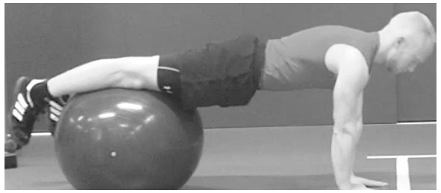	

Elite level (2x8-12 repetitions)			
**F**	Starting position as in 4E<br><br>Flex both elbows, followed by a plyometric push-up with a lift-off of both hands at the end of the movement<br><br>Make sure to control the landing and remain stable around scapula (eccentric work)<br><br>Repeat	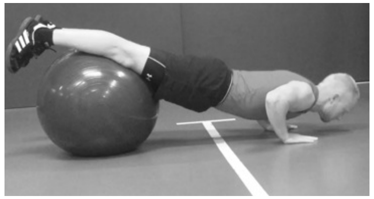<br>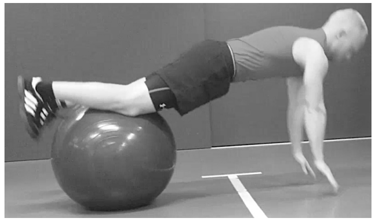	
**G**	Prone lying with arms in shoulder width<br><br>Extend both arms with body straight (lower back in neutral)<br><br>Lower the body to the floor, perform a plyometric push-up followed by a double lift-off (hands and feets lifted simultanoesuly from the ground)<br><br>Make sure to control the landing and remain stable around scapula (eccentric contraction)<br><br>Repeat<br><br>If further progression is needed:<br>Use of theraband (one end fixed to a wall, other end placed in the hand of the injured shoulder) during the double lift-off to resist horizontal abduction in affected shoulder	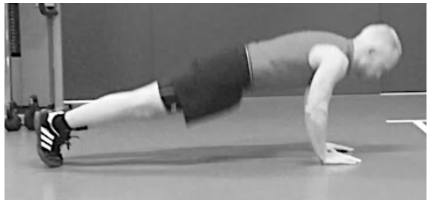<br>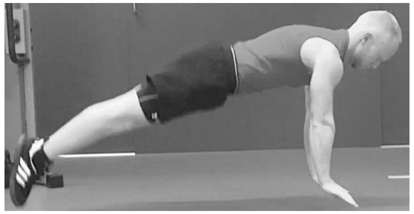	

Exercise 5	Dynamic glenohumeral muscle stability

Biomechanical purpose: To optimize the dynamic stability in the shoulder while keeping scapula and glenohumeral joint in control

Purpose from a patient perspective: *”This exercise is important to improve your ability and skills during sudden shoulder movements – as often experienced during sports activities*

Basic level (3x10 seconds)			
**Level**	**Performance**	**Photos**	**Feedback to the patient**
**A**	Sitting erect on an exercise ball with both feet at the ground<br>Neck and lower back in neutral<br>Place a folded towel between the elbow and the abdomen<br>Set the glenohumeral joint and scapula<br><br>Perform isometric contractions with resistance from the opposite hand	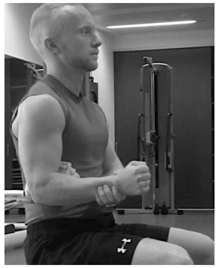	Avoid excessive shoulder elevation during the exercise
**B**	Fix one end of a theraband at hand height and place the other end of the theraband in the hand at the injured side<br><br>Starting position as in A<br><br>Tighten the theraband<br><br>Perform small dynamic, rhythmic contractions while keeping scapula and the glenohumeral joint stable, relax, repeat	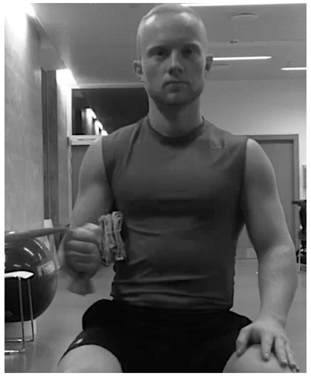	As above

**C**	Starting position as in 5B<br><br>Lift the leg at the injured side from the ground<br><br>Tighten the theraband<br><br>Perform small dynamic, rhythmic contractions while keeping scapula and the glenohumeral joint stable, relax, repeat	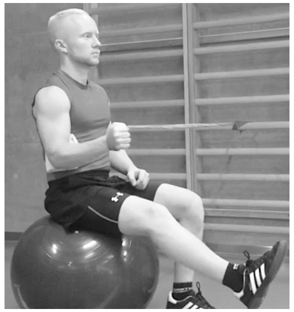	As above
**D**	Starting position as in 5B<br><br>Abduct the shoulder 40 degrees in the scapular plane, flex the elbow to 90 degrees<br><br>Tighten the theraband<br><br>Perform small dynamic, rhythmic contractions while keeping scapula and the glenohumeral joint stable, relax, repeat	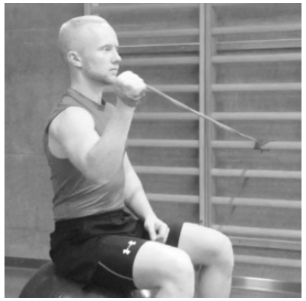	As above
E	Standing with feets in hip distance apart<br><br>Arms down<br>Tighten a theraband between both hands<br><br>Perform small dynamic, rhythmic contractions to keep the theraband tight while moving both arms up above the head and down again<br><br>Relax, repeat	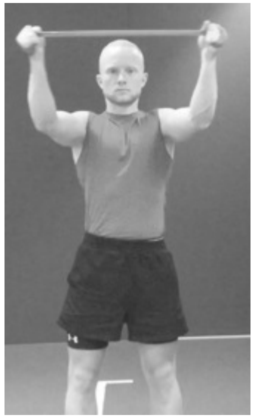	As above<br><br>Keep the core muscles stable

Elite level (3x20 seconds)			
**F**	Fix one end of a theraband behind the body in shoulder height<br>Fix a bottle of water near the end of the theraband close to the hand of the injured hand<br><br>Standing with the leg opposite to the injured shoulder in front, knees slightly flexed, bodyweight transferred to the front leg (front knee aligned with the hip and foot)<br>Upper and lower back positioned in neutral<br><br>Place the shoulder above 90 degrees of abduction (throwing position), elbow flexed, shoulder in neutrol rotation (hand against the ceiling)<br><br>Set the glenohumeral joint and scapula<br><br>Perform small dynamic, rhythmic contractions keeping the theraband tight and remain scapula and the glenohumeral joint stable, relax, repeat	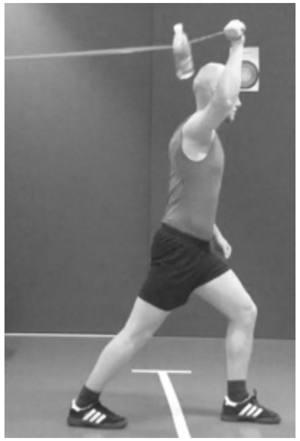	As above
**G**	As in 5F<br><br>Lift the leg at the injured side from the ground<br><br>If further progression is needed:<br>Close the eyes, increase shoulder abduction, increase the resistance from the theraband	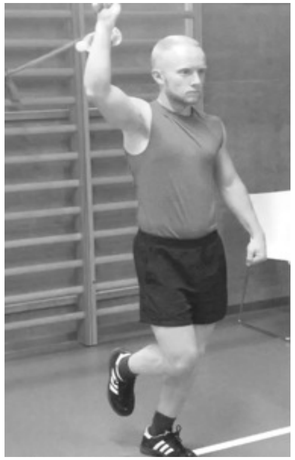	As above

Exercise 6	Glenohumeral proprioception (use of exercise ball)

Biomechanical purpose: To optimize shoulder joint position sense

Purpose from a patient perspective: *”This exercise is important to improve your body´s (and shoulder) awareness of where it is in nature. These skills are important to return to pre-injury performance levels.*

Basic level (2x5 repetitions)

**Level**	**Performance**	**Photos**	**Feedback to the patient**
**A**	Lying supine with knees flexed<br><br>Place an exercise ball on the palms of both hands with elbows flexed<br>Extend both elbows while balancing the ball on both hands.<br>Lower both arms again, relax, repeat<br><br>The goal of the exercise is to be able to perform the exercise five times in a row without dropping the exercise ball	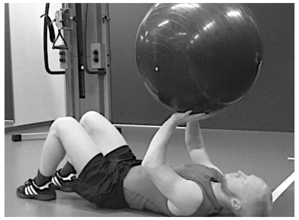 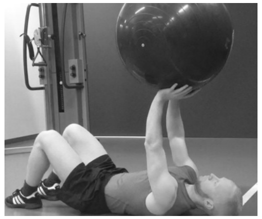	Move slowly<br><br>If the bold drops, let it go, do not attempt to catch it
**B**	As in 6A – though, with eyes closed<br><br>The goal of the exercise is to be able to perform the exercise five times in a row without dropping the exercise ball	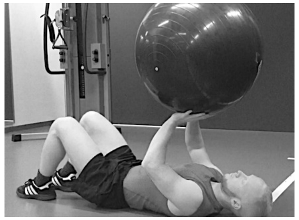 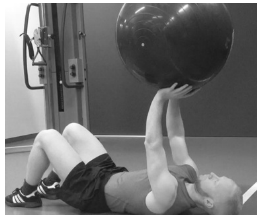	As above

**C**	Starting position as in 6A<br><br>Place an exercise ball on the palm of the injured side, elbow flexed<br>Keep the contralateral hand close to the exercise ball for support<br>Extend the arm of the injured side while balancing the ball on the palm<br>Lower the arm, relax, repeat<br><br>The goal of the exercise is to repeat the exercise five times in a rowwithout the need of support from the contralateral hand and without dropping the exercise ball	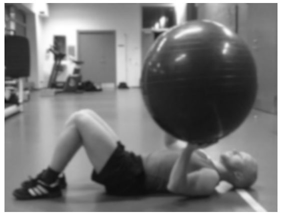 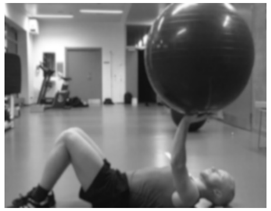	As above<br><br>Keep the non-injured arm close to the exercise ball for support and to avoid sudden drop of the exercise ball
**D**	As in 6C<br><br>With the injured arm fully extended and balancing the exercise ball, slowly move the arm backwards behind the head, return again, lower the arm to starting position, relax, repeat<br><br>The goal of the exercise is to repeat the exercise five times in a row without the need of support from the contralateral hand and without dropping the exercise ball	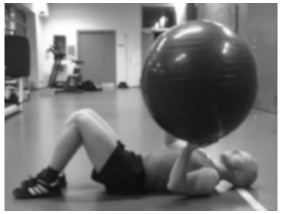 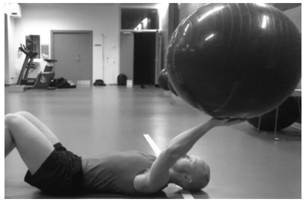	As above
**E**	Standing with the leg opposite to the injured shoulder in front, knees slightly flexed, bodyweight transferred to the front leg (front knee aligned with the hip and foot)<br>Upper and lower back positioned in neutral<br><br>Flex the elbow at the injured side to 90 degrees, place an exercise ball on the palm of the injured side<br><br>Extend the arm, when fully extended, perform a small rectangular movement with the arm, while balancing the exercise ball,<br>Return the arm to starting position, relax, repeat<br><br>The goal of the exercise is to repeat the exercise five times in a row without the need of support from the contralateral hand and without dropping the exercise ball	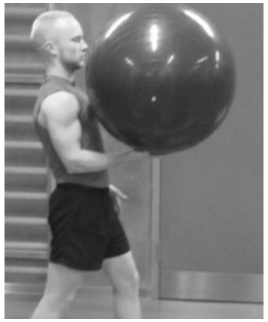 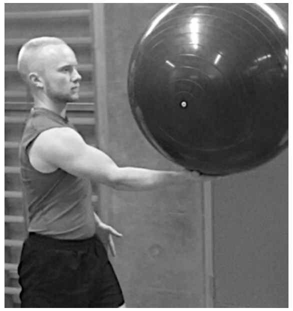	As above

Elite level (2x10 repetitions)			
**F**	As in 6E – though, with eyes closed<br><br>The goal of the exercise is to be able to perform the exercise ten times in a row without dropping the exercise ball	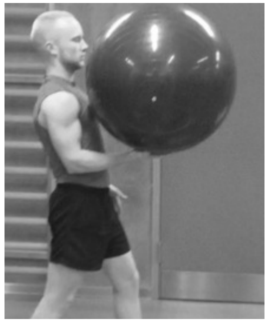 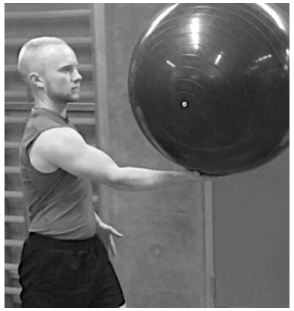	As above
**G**	Starting position as in 6E<br><br>While balancing the exercise ball, perform a lunge exercise with the contralateral leg in front (knee in alignment with hip and foot) simultanoesuly extending the arm in a forward direction<br><br>Return to starting position, relax, repeat<br><br>The goal of the exercise is to be able to perform the exercise ten times in a row without dropping the exercise ball<br><br>If further progression is needed:<br>From the lunge position, perform an explosive return to the starting position, closed eyes	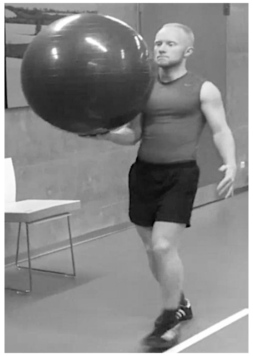 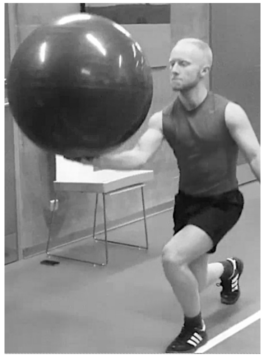	As above

Exercise 7	Glenohumeral proprioception (use of laser pointer)

Biomechanical purpose: To optimize shoulder joint position sense

Purpose from a patient perspective: *”This exercise is important to improve your body´s (and shoulder) awareness of where it is in nature. These skills are important to return to pre-injury performance levels.*

Basic level (2x5 repetitions)			
**Level**	**Performance**	**Photos**	**Feedback to the patient**
**A**	Fix a shooting target (with a centered bullseye) at hip height at a wall<br>Fix a laser pointer at the wrist of the injured arm with the dot pointing away from the body<br><br>Standing (with feets in hip distance apart) with one arm length in addition to one foot length away from the wall, arms resting<br><br>Set the glenohumeral joint and scapula<br><br>Move the injured arm in forward flexion, elbow extended, to center the laser dot at bullseye<br>Close the eyes, count to three<br>Return the arm to starting position<br>Re-position the arm, elbow extended, so that the laser dot targets the bullseye<br><br>Open the eyes to check the exercise accuracy<br><br>The goal of the exercise is to be able to position the laser dot as close to bullseys five times in a row	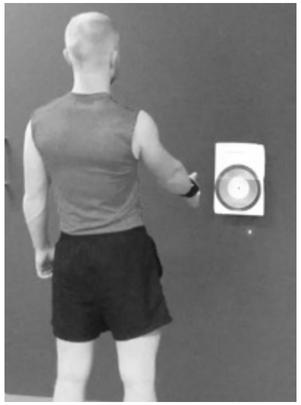	Avoid excessive shoulder elevation during shoulder flextion
**B**	Fix a shooting target (with a centered bullseye) at shoulder height<br><br>Perform the exercise as in 7A	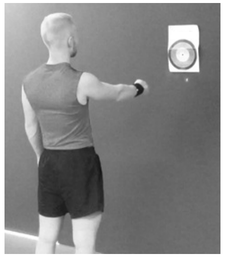	As above

**C**	Fix a shooting target (with a centered bullseye) above head height<br>Perform the exercise as in 7A	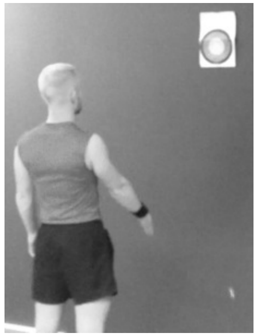	As above
**D**	Fix a shooting target (with a centered bullseye) above head height<br>Standing in tandem position with the contralateral leg in front<br>Perform the exercise as in 7A	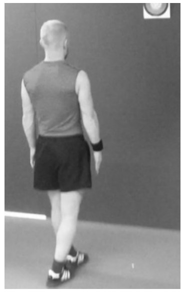	As above
**E**	Fix a shooting target (with a centered bullseye) above head height<br>Standing with the leg at the injured side liftet from the ground<br>Perform the exercise as in 7A	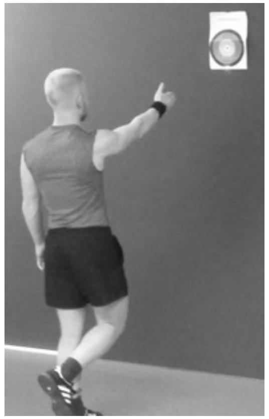	As above

Elite level (2x10 repetitions)			
**F**	Fix a shooting target (with a centered bullseye) above head height<br><br>Place a bottle of water in the hand of the injured side<br><br>Standing with the leg at the injured side liftet from the ground<br><br>Perform the exercise as in 7A<br><br>The goal of the exercise is to be able to position the laser dot as close to bullseys ten times in a row	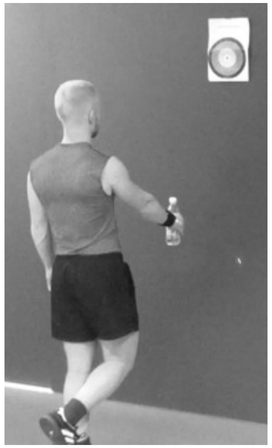	As above
**G**	Fix a shooting target (with a centered bullseye) above head height<br><br>Place one end of a theraband underneath the foot opposite to the injured shoulder and the other end in the hand of the injured shoulder<br><br>Perform the exercise as in 7A<br><br>The goal of the exercise is to be able to position the laser dot as close to bullseys ten times in a row<br><br>If further progression is needed:<br>Increase the speed of the exercise, fix the shooting target higher up the wall	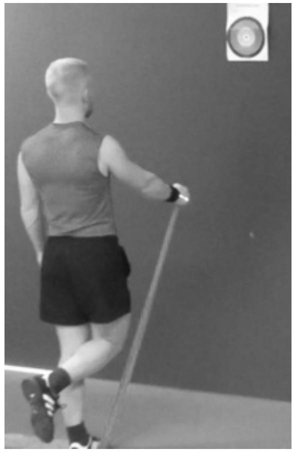	As above

References

1. Magarey, M.E. and M.A. Jones, *Dynamic evaluation and early management of altered motor control around the shoulder complex.* Man Ther, 2003. **8**(4): p. 195-206.

2. Karatsolis, K., S. Athanasopoulos, *The role of exercise in the conservative treatment of the anterior shoulder dislocation.* Journal of Bodywork and Movement Therapies, 2006. **10**: p. 211-219.

3. Jaggi, A. and S. Lambert, *Rehabilitation for shoulder instability.* Br J Sports Med, 2010. **44**(5): p. 333-40.

4. Hess, S.A., *Functional stability of the glenohumeral joint.* Man Ther, 2000. **5**(2): p. 63-71.

5. Myers, J.B. and S. Oyama, *Sensorimotor factors affecting outcome following shoulder injury.* Clin Sports Med, 2008. **27**(3): p. 481-90, x.

6. Myers, J.B., C.A. Wassinger, and S.M. Lephart, *Sensorimotor contribution to shoulder stability: effect of injury and rehabilitation.* Man Ther, 2006. **11**(3): p. 197-201.

7. Lephart, S.M., et al., *Proprioception of the shoulder joint in healthy, unstable, and surgically repaired shoulders.* J Shoulder Elbow Surg, 1994. **3**(6): p. 371-80.

8. Wilk, K.E., L.C. Macrina, and M.M. Reinold, *Non-operative rehabilitation for traumatic and atraumatic glenohumeral instability.* N Am J Sports Phys Ther, 2006. **1**(1): p. 16-31.

9. Wilk, K.E., C.A. Arrigo, and J.R. Andrews, *Current concepts: the stabilizing structures of the glenohumeral joint.* J Orthop Sports Phys Ther, 1997. **25**(6): p. 364-79.

10. Stensrud, S., E.M. Roos, and M.A. Risberg, *A 12-week exercise therapy program in middle-aged patients with degenerative meniscus tears: a case series with 1-year follow-up.* J Orthop Sports Phys Ther, 2012. **42**(11): p. 919-31.

11. Eitzen, I., et al., *A progressive 5-week exercise therapy program leads to significant improvement in knee function early after anterior cruciate ligament injury.* J Orthop Sports Phys Ther, 2010. **40**(11): p. 705-21.

12. Frobell, R.B., et al., *A randomized trial of treatment for acute anterior cruciate ligament tears.* N Engl J Med, 2010. **363**(4): p. 331-42.

13. Ageberg, E., A. Link, and E.M. Roos, *Feasibility of neuromuscular training in patients with severe hip or knee OA: the individualized goal-based NEMEX-TJR training program.* BMC Musculoskelet Disord, 2010. **11**: p. 126.

14. Christiansen, D.H., et al., *Physiotherapy after subacromial decompression surgery: development of a standardised exercise intervention.* Physiotherapy, 2015. **101**(4): p. 327-39.

15. Holmgren, T., et al., *Effect of specific exercise strategy on need for surgery in patients with subacromial impingement syndrome: randomised controlled study.* Br J Sports Med, 2014. **48**(19): p. 1456-7.
